# Molecular determinants of differential substrate selection between the Src family kinases Lck and Src

**DOI:** 10.3389/fimmu.2026.1880421

**Published:** 2026-08-10

**Authors:** Konstantina Karpouzou, Marco D’Abramo, Alessandro Grottesi, Oreste Acuto, Konstantina Nika

**Affiliations:** 1 Department of Biochemistry, School of Medicine, University of Patras, Patras, Greece; 2 Department of Chemistry, University of Rome “La Sapienza”, Rome, Italy; 3 SuperComputing Applications and Innovation Department, CINECA - Italian Computing Centre, Rome, Italy; 4 Cell Signaling Laboratory, Sir William Dunn School of Pathology, University of Oxford, Oxford, United Kingdom

**Keywords:** ITAM phosphorylation, Lck, Src, substrate specificity, T cell receptor signaling

## Abstract

Src family kinases (SFKs) share highly conserved catalytic domains yet display distinct biological functions, raising the question of how substrate specificity is achieved. Here, we investigate the basis of differential ITAM recognition by Lck and Src, using complementary cellular and computational approaches. In-cell assays demonstrated that, contrary to Lck, Src was completely incapable of phosphorylating the TCR ITAMs when ectopically expressed in a T cell environment. Domain-swapping experiments further revealed that substitution of the Src kinase domain with that of Lck was sufficient to confer ITAM phosphorylation and trigger downstream TCR signaling responses, whereas exchange of adaptor domains had minimal effect. To gain structural insight into this selectivity, molecular dynamics simulations followed by ensemble docking were performed using conformations sampled from simulations of the Lck and Src kinase domains together with an ITAM peptide. The analyses revealed that Lck consistently accommodates the ITAM peptide with higher shape complementarity than Src, supporting a more favorable structural environment for ITAM recognition. Our findings demonstrate that subtle differences within the kinase domain of closely related SFKs can profoundly influence substrate recognition, with significant consequences for signaling responses in living cells.

## 1 Introduction

Src family kinases (SFKs) are indispensable transducers of signaling responses, serving a wide variety of receptors and controlling a plethora of functions such as survival, regulation of cell growth, activation, and motility. The best-characterized family members, c-Src, Fyn, Lck and Lyn are amongst the most extensively studied kinase proto-oncogenes and constitute very attractive therapeutic targets due to their central roles in several diseases (1).

Members of the SFK family share a common structural architecture. They contain a unique domain, the extreme N-terminus (SH4 domain) of which, can undergo lipid modifications, an SH3 and an SH2 domain with adaptor functions, a regulatory linker sequence followed by the kinase domain, and a C-terminal inhibitory tail (2). The enzymatic function of all SFK members is regulated through a highly conserved mechanism, involving specific conformational changes (2). Intramolecular interactions between the SH2 domain and phosphorylated Y527 (amino-acid numbering based on the archetype of SFKs, c-Src), within the C-terminal inhibitory tail, endorse a so-called “closed” conformation, which prevents substrate accessibility to the catalytic pocket. It has been proposed that the closed conformation is further stabilized through the interaction of the SH3 domain with the regulatory linker. Release of these intramolecular blocks by dephosphorylation of Y527 and/or the active displacement of the SH2/SH3 domains by binding interactors, forces the kinase into a functional open conformation, known as the “primed” form. However, optimal enzymatic activity requires the repositioning of a loop inside the kinase domain, which is achieved via trans-autophsophorylation of Y416. Consequently, the enzymatic activity of SFKs is tuned by a dynamic regulation on the phosphorylation status of the two regulatory tyrosines, Y416 and Y527. Two enzymes with opposing actions have been extensively characterized as the major rheostats of SFK activity: the kinase Csk, responsible for phosphorylating Y527, and the transmembrane phosphatase CD45 (in haematopoietic cells) (1, 2).

SFKs are a highly conserved family with 60-90% sequence identity in their catalytic sites and very similar 3D structures (2). Accordingly, high-throughput screening analyses (3–6) have demonstrated significant, yet not absolute, overlap amongst SFK substrates. Determinants of selectivity have been attributed to tissue-restricted expression and unique patterns of subcellular localization, but importantly, also structural features that influence substrate selection and effective catalysis. To revisit the question of SFK selectivity, beyond the conventional *in vitro* settings, we have developed an experimental system that enables monitoring SFK-substrate interactions within a physiological cellular environment. We selected Lck and c-Src for in-cell substrate specificity comparisons. We reasoned that intrinsic determinants of selectivity would be more clearly resolved by contrasting a T cell–specialized kinase (Lck) with the ubiquitously expressed prototypical SFK c-Src, which is largely absent from resting peripheral T cells (7), than by comparing immune-restricted SFKs such as Lck, Fyn, Lyn, or Hck.

Our data show that, within an intact cellular environment Src and Lck can display an astonishing ability for substrate discrimination and that this behavior is highly dependent on determinants located within their kinase domains. Identification of such features can provide a framework for future studies aimed at developing selective and clinically safe SFK inhibitors, a goal hindered by poor isoform selectivity (8, 9).

## 2 Results

The central role of Lck in initiating TCR signalling is well established, with the CD3 and ζ chain ITAMs (Immunoreceptor Tyrosine-based Activating Motifs) constituting its primary and most-extensively characterized substrates (10). We therefore selected the T-cell cellular environment where, unlike Src, Lck has been evolutionary selected to operate, to assess substrate selectivity, aspiring to record differences in ITAM-phosphorylating potencies between the two SFKs.

**Figure 1:**
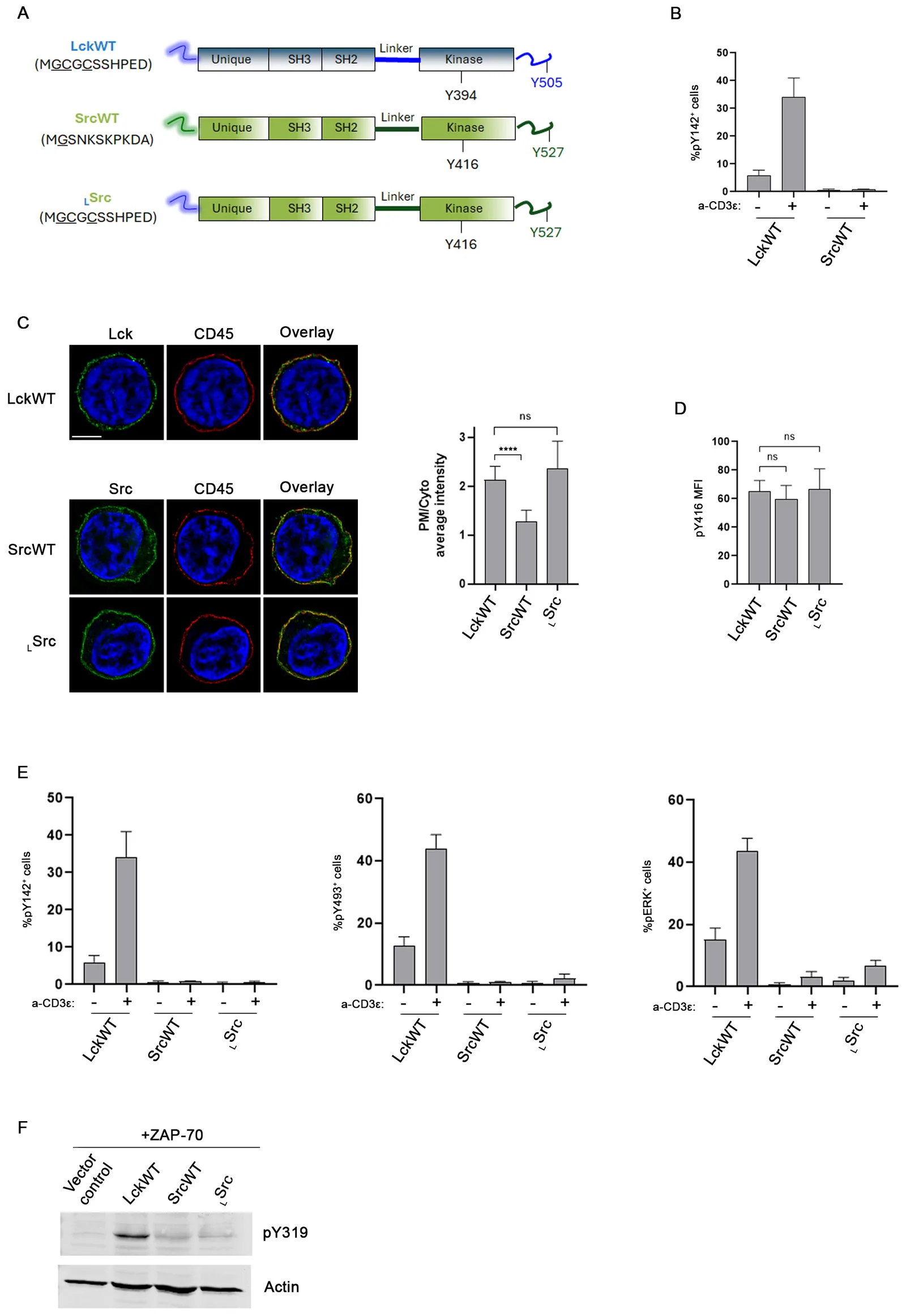
Differential substrate recognition by Lck and Src. **(A)** Schematic diagrams of Lck and Src domain architecture. Functional domains and regulatory tyrosines of Lck and Src are labelled and color-coded. The SH4 domain amino acid sequences are listed, myristoylation (Glycine) and palmitoylation (Cysteine) sites are underlined. **(B)** SrcWT is unable to induce ITAM phosphorylation in intact T cells. JCaM1.6 cells expressing Lck or Src wild-type were either left untreated or stimulated with 1μg/ml a-CD3ϵ (clone UCHT1) for 2min at 37°C and stained with a-pY142 and a-pY416. Graph shows the percentage of cells with levels of pY142 exceeding the -Dox baseline, within pY416^+^-gated populations (gating strategy described in **Supplementary Figure 1**). Data from four independent experiments. **(C)** SH4 domain exchange redirects Src subcellular distribution to mimic that of Lck. SIM images of JCaM1.6 cells expressing LckWT, SrcWT or _L_Src stained with anti-CD45 (red) and either anti-Lck or anti-Src (green) as indicated. Nuclei were counterstained with DAPI (blue). Adjacent graphs display the PM/Cyto ratio of average intensity fluorescence for each antibody. Number of cells ≥20 from 2 independent experiments. Scale bar: 5μm. **(D)** SH4 domain exchange does not compromise the levels of active Src. MFI values of the same cell lines as in **(B)** stained with a-pY416 and analysed by FACS (Gating strategy described in **Supplementary Figure 1**). Pooled data from 4 independent experiments. **(E)** Differential subcellular distribution does not account for the lack of ITAM phosphorylation by Src. Same cell lines as in **(B)**, were either left untreated or stimulated with 1μg/ml a-CD3ϵ for 2min at 37°C. Samples were stained with a-pY142, a-pY493, (recognizing the phosphorylated form of the activating ZAP-70 Y493) or a-pERK, (detecting the doubly phosphorylated active status of the ERK1 and ERK2 MAPKs). Graphs display percentage of cells with phosphoantibody fluorescence levels exceeding the -Dox baseline, within pY146^+^-gated populations. Pooled data from 4 independent experiments. **(F)** Differential recognition between Lck and Src extends to ZAP-70 Y319. HEK293 cells were transiently transfected with ZAP-70 together with empty vector, LckWT, SrcWT or _L_Src. Total cell lysates were analyzed by immunoblotting using an antibody against the phosphorylated form of Y319 of ZAP-70 (pY319). Actin expression (lower panel) is shown as a loading control. Representative western blots from 2 independent experiments. Comparable levels of activity amongst the transfected SFK constructs are shown in **Supplementary Figure 1D**. Unpaired Student t test; mean +/- SD; ns, not significant. Unpaired Student t test; mean +/- SD; ns, not significant, ****P < 0.0001.

Wild-type human Lck and Src were expressed in the Lck-deficient Jurkat variant JCaM1.6 via lentiviral gene transfer and using a tetracycline-dependent transcriptional activator. This inducible gene expression system has been previously characterized by our group [(11, 12) and **Supplementary Figure 1**]. Lck- or Src-expressing cells were stimulated with anti-CD3ϵ for 2min at 37°C and stained with anti-pY142, an antibody specifically recognizing the phosphorylated form of the second tyrosine on the third ITAM of the TCRζ chain (**Figure 1B**), a well-recognized Lck direct substrate. To quantify induction of Y142 phosphorylation by the individual kinases, samples were co-stained with a-pY416; this antibody indiscriminately recognizes the phosphorylated form of the activation loop regulatory tyrosines (Y394 in Lck and Y416 in Src). Since the antibody epitope covers a sequence that is absolutely conserved in all SFKs (2), it allows measurements of pY142 within gates of equal levels of SFK activity (gating strategies described in **Supplementary Figure 1**). Collective FACS analysis data (**Figure 1B**) revealed that unlike Lck, Src was completely incapable of inducing Y142 phosphorylation.

The SH4 domain of Lck is a major determinant for its subcellular distribution and lateral residency within distinct plasma membrane (PM) compartments (12, 13). Acyl group modifications within this short 10 amino acid N-terminal sequence, include co-translational myristoylation of Gly2 and the post-translational palmitoylation of Cys3 and Cys5, which appear to be essential for Lck-mediated TCR signalling responses (14, 15). Src has been shown to partly reside at the PM but also to perinuclear membranes, and in cytoplasm-based vesicles. Similarly to Lck, SrcSH4 is myristoylated on Gly2 but lacks the palmitoylation signals, instead, it contains a polybasic region involved in membrane association (14, 16). Despite their tendency for PM residency, two-colour PALM microscopy of fluorescently-labelled LckSH4 and SrcSH4 showed no significant overlap between these two constructs at the surface of transfected cells, indicating distinct PM compartmentalization (17). To exclude that Src inefficiency in promoting Y142 phosphorylation was due to its subcellular distribution that might prohibit proximity to the TCR complex, we created a chimeric Src construct (_L_Src) in which the SrcSH4 domain was substituted by the corresponding sequence of Lck (**Figure 1A**).

Cells expressing LckWT, SrcWT or _L_Src were analysed by Structured Illumination Microscopy (SIM) (**Figure 1C**). CD45 and DAPI staining served as a means of defining ROIs representing the PM and nucleus respectively. Measurements of the average fluorescence, obtained by anti-Lck or anti-Src staining, within ROIs that defined the PM and cytoplasmic compartments were utilized to calculate ratios of PM versus cytoplasmic localization (PM/Cyto) for the specific constructs. As previously reported, LckWT primarily resided at the plasma membrane, with some anti-Lck fluorescence also being detected intracellularly, whereas SrcWT was proportionally distributed between the PM and cytoplasmic compartments. _L_Src displayed enhanced PM localization compared to SrcWT, with a profile mimicking that of Lck (**Figure 1C**). Misplacement of Src from its natural cellular residence did not sizably affect the phosphorylation of Y416. Indeed, all three SFK constructs appeared to have comparable levels of their active form, as revealed by quantitative FACS using the anti-pY416 antibody (**Figure 1D**).

However, despite residing primarily in the same cellular compartment as Lck and the TCR complex, _L_Src was unable to phosphorylate the ζ chain Y142, and ignite downstream phosphorylation events directly or indirectly controlled by Lck enzymatic activity (**Figure 1E**).

Collectively, these data indicate that Src’s inability to functionally replace Lck is due neither to compromised substrate proximity, nor to inadequate levels of the kinase active form. Rather, it is deduced that intrinsic structural features of Lck define its unique ability to accommodate the TCR ITAMs for phosphotransfer.

To further substantiate substrate preferences between Lck, Src and _L_Src, we assessed their catalytic potency against an independent well-characterized Lck substrate, Y319 of ZAP-70 (18, 19). To exclude that any potential inability of Src or _L_Src to phosphorylate Y319 would merely reflect the absence of kinase-substrate proximity resulting from lack of ITAM phosphorylation (and the consequent lack of ZAP-70 recruitment), ZAP-70 was transiently co-expressed together with Lck, Src or _L_Src in HEK 293 cells and Y319 phosphorylation was assessed by western blotting (**Figure 1F**). Unlike Lck, neither SrcWT nor _L_Src induced appreciable Y319 phosphorylation. These findings provide independent cellular evidence that the differential substrate recognition described in our study is not confined to a single TCR ITAM tyrosine.

**Figure 2:**
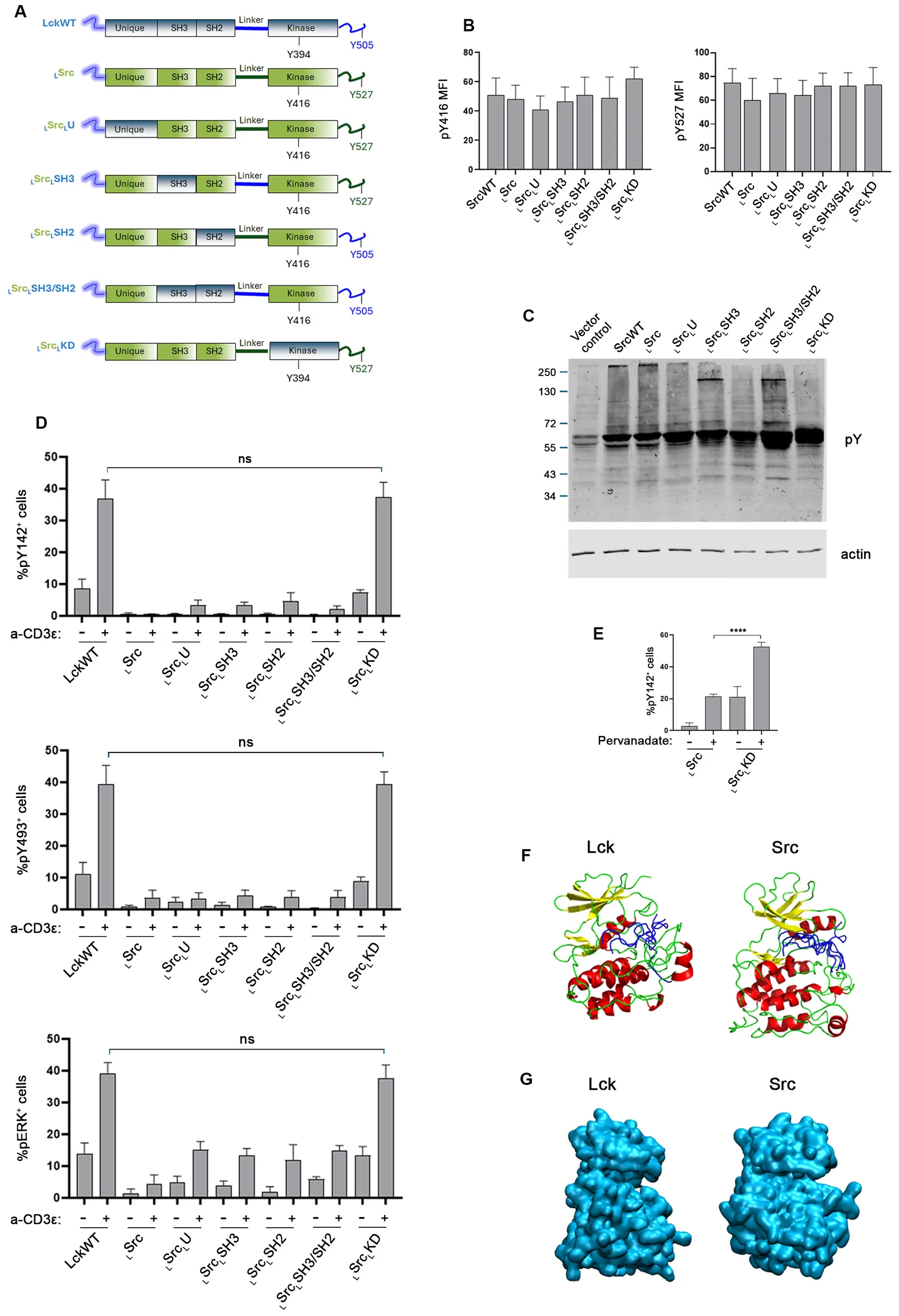
Substrate discrimination features are encoded in the Lck KD. **(A)** Design of the Src domain-swap chimeras. Schematic diagrams of Src chimeric proteins. The functional domains and regulatory tyrosines of Lck and Src are designated and color-coded. **(B)** Domain swapping did not compromise the conformational integrity of Src. JCaM1.6 cells expressing SrcWT or chimeric forms were stained with a-pY416 or a-pY527 and analysed by FACS. MFI values of each antibody are displayed in the corresponding graphs as indicated. Pooled data from 4 independent experiments. Statistical analysis did not identify significant differences between SrcWT and the indicated chimeric constructs. Individual statistical annotations have been omitted to improve figure readability. **(C)** Domain swap did not compromise Src catalytic activity against endogenous substrates. HEK293 cells were transiently transfected with SrcWT, _L_Src or the domain-swap chimeras as indicated. Total cell lysates were analyzed by western blot using the anti-phosphotyrosine antibody 4G10 (pY), to demonstrate the ability of the Src constructs to induce increases in global tyrosine phosphorylation of endogenous proteins. Actin immunoblot (lower panel) serves as a loading control. Representative western blots from 2 independent experiments. Comparable levels of activity amongst the transfected SFK constructs are shown in **Supplementary Figure 2A**. **(D)** Incorporation of the Lck kinase domain into Src rescues ITAM phosphorylation and TCR signaling responses. JCam1.6 cells expressing LckWT or the indicated Src chimeras were stimulated, stained and analysed as in **Figure 1E**. Graphs display pooled data from 4 independent experiments. Statistical analysis was performed for all indicated groups. Only the comparison between LckWT and _L_Src_L_KD stimulated samples is annotated in the graphs, as it represents the biologically relevant assessment of TCR signaling rescue. Statistical analysis between Lck and the chimeric constructs consistently yielded P < 0.0001; these annotations have been omitted from the graph for clarity. **(E)** Pervanadate treatment fails to restore the ITAM phosphorylation burden imposed by the Src kinase domain. Cells expressing _L_Src or _L_Src_L_KD were either left untreated or incubated with 100μM Pervanadate for 10min at 37°C, stained with a-pY142 and a-Src and analysed by FACS. Graph shows the percentage of cells with levels of pY142 exceeding the -Dox baseline, within Src^+^-gated populations. Data from four independent experiments. **(F, G)** Intrinsic structural features of the Lck Kinase domain enable optimal ITAM accommodation. **(F)** Lck- and Src-ITAM complexes as obtained by docking calculations (see Methods). The Lck and Src KDs are represented as ribbon and the ITAM peptides as tube (blue marine). **(G)** Solvent accessible surface X-ray crystal structures of the kinase domains of Lck and Src (both represented in light blue) showing the location of a crevice located between the C- and N-lobe of the kinase. Unpaired Student t test; mean +/- SD; ns, not significant, ****P < 0.0001.

To pinpoint the Lck determinants that would provide substrate specificity, we took advantage of the highly conserved structural architecture of SFKs. Towards this end, we generated a series of Lck-Src chimeras in which individual functional domains of Src were sequentially substituted by the corresponding ones of Lck (**Figure 2A**; **Supplementary Table 1** for a detailed description). All chimeras were produced on the _L_Src background to ensure no bias due to divergent subcellular distributions of the chimeric constructs. To preserve the intramolecular interactions responsible for regulating SFK activity, swaps of the SH3 and SH2 domains, were coupled with corresponding substitutions of the linker and C-terminal inhibitory tail sequences, respectively (**Figure 2A**; **Supplementary Table 1**). Chimeric Src proteins were individually expressed in the JCaM1.6 line. The swap of functional domains did not alter the phosphorylation levels of the regulatory tyrosines (Y416 and Y527: the inhibitory C-terminal tyrosine of Src), nor the ability of the Src chimeras to phosphorylate endogenous substrates in HEK293 cells (**Figures 2B, C**; **Supplementary Figure 2A**). This indicated that incorporation of the corresponding Lck domains did not disrupt Src kinase conformation or regulation. To assess whether Lck functional domains might rescue TCR signalling responses, cell lines expressing the chimeric Src constructs alongside LckWT were subjected to anti-CD3 stimulation experiments, (**Figure 2D**). Incorporation, within the Src protein, of the Lck Unique domain, SH3, SH2 or a combination of the latter two (SH3/SH2) induced somewhat improved yet severely compromised responses and only at very high levels of Src kinase activity (**Supplementary Figure 2B**). This pattern changed only when the Src kinase domain (KD) was replaced by the corresponding one of Lck. These data indicate that there are determinants within the Lck kinase domain playing a major part in substrate selectivity towards the ITAMs of the TCR.

To further substantiate the concept that the Src KD is non-optimally configured for accommodating Lck-restricted substrates, we treated the _L_Src and _L_Src_L_KD lines with pervanadate. By inhibiting tyrosine phosphatases, pervanadate drives widespread ITAM and global tyrosine phosphorylation in T cells, effectively bypassing the normal regulatory constraints of the TCR signalling machinery. Although ITAM phosphorylation was clearly enhanced in _L_Src cells following pervanadate treatment (when compared with a-CD3 stimulations, **Figure 2D**), the levels remained substantially low; notably, they only reached baseline phosphorylation levels recorded in untreated _L_Src_L_KD cells (**Figure 2E**).

The finding that replacement of the Lck KD largely accounted for ITAM phosphorylation by Src, prompted us to investigate whether intrinsic structural properties within the Lck and Src KDs could have a role in distinct substrate recognition. To this end, Molecular Dynamic (MD) simulations were performed using the crystallographic structures of the kinase domains of Lck (PDB: 3LCK) (20) and Src (PDB: 1YI6) (21) in their active conformations (with phosphorylated activation loop tyrosines Y394 and Y416 respectively) and the NMR-derived structure of an ITAM peptide (PDB: 1CV9) (22), using the Patchdock suite. The total simulation time for both systems was 150 ns, and approximately one frame out of 150 ps of simulation time were selected, resulting in about 1000 docking experiments. The resulting docking solutions were evaluated using three complementary metrics: i) pose score, based on shape complementarity; ii) pose convergence, based on clustering methods; and iii) burial depth measures. The analyses consistently indicated that the MD-derived conformational ensemble of Lck accommodates the ITAM peptide more favourably than that of Src (**Figure 2F**). In particular, the PatchDock geometric score based on shape complementarity for Lck is on average 6% higher than for Src. Furthermore, the pose clustering resulted in 89% of all poses in the main docking site as compared to 62% in Src. Regarding the burial depth analysis, although both receptors engage a similar number of residues (about 30 each), in Lck those contacts wrap around the ITAM peptide more extensively, i.e., +12% denser atomic packing and greater solvent shielding (**Table 1**). The best 5 poses according to the Patchdock score were superimposed onto the Lck/Src KDs (**Figure 2G**). In agreement with the burial metric described above, the representative Lck docking poses occupy more deeply buried positions within the substrate-binding region, whereas the corresponding Src poses are located in a more solvent exposed region.

**Table 1:** Shape complementarity scores.

KD	Mean	Median	Std	Min	Max
Lck	8851	8801	665	6418	11172
Src	8315	8227	697	6840	10752

## 3 Discussion

This work presents a new mechanistic insight into the substrate preference of SFKs. Over the past decades, *in vitro* assays with short synthetic peptides, high-throughput *in vitro* screens using Surface Plasmon Resonance-based profiling of hundreds of substrates, as well as large-scale peptide library screens combined with next-generation sequencing (3–6, 22), have revealed that SFKs share a large pool of common substrates, indicating widespread redundancy and functional overlap. Our data demonstrate that when expressed in their full-length form within an intact cellular environment, Lck and Src exhibit remarkable selectivity towards the TCR ITAMs and ZAP-70 Y319 and in the initiation of downstream signaling cascades.

Rigorous examination of features within the Src structure, that prohibit ITAM recognition and phosphorylation, excluded potential involvement of its, differential to Lck, subcellular distribution and thus proximity to its substrates. The adaptor domains of Lck and Src play a critical role in shaping their catalytic output by mediating and/or stabilizing selective interactions with specific substrates (23–25). Notably, the SH2 domain of Lck can interact with anionic PM lipids, thereby providing an additional layer of spatiotemporal regulation for the recruitment of substrates such as the TCRζ chain and ZAP-70 (23, 24). However, substitution of Src SH3 and SH2 (individually or in combination) with the corresponding domains of Lck, resulted in very modest enhancement of Y142 phosphorylation. Moreover, this effect was observed only when the Src chimeras were expressed at very high levels. In fact, only incorporation of the Lck kinase domain, bestowed to Src the ability to phosphorylate the TCR ITAMs and trigger downstream signalling responses, highlighting intrinsic selectivity at the catalytic level.

MD-derived ensemble docking analyses suggest that differences in the conformational properties of Lck and Src kinase domains may contribute to differential ITAM recognition. While the global fold of Lck and Src kinase domains is highly similar, across the MD-derived conformational ensembles, Lck exhibited improved shape complementarity and deeper peptide burial than Src, consistent with a greater ability to accommodate the ITAM peptide. These computational observations provide a plausible structural rationale for the in-cell observed differences in substrate recognition. Our MD-derived ensemble docking results are in good agreement with the models proposed by Gaul et al. (22) and Shah et al. (3), supporting a binding mode in which the ITAM peptide engages the cleft between the N- and C-lobes. Notably Shah et al. report that Lck and Src exhibit subtle sequence preferences particularly with respect to electrostatic determinants; Src favors negatively charged substrates, whereas Lck preferentially phosphorylates substrates enriched in positively charged residues. Our findings complement these observations by suggesting that electrostatic determinants operate within a structural context in which the conformational properties of the Lck kinase domain provide more favorable accommodation of the ITAM peptide than those of Src. In this context, the Lck kinase domain appears better suited for ITAM phosphorylation. Although our computational analyses do not establish the definitive molecular mechanism underlying substrate selectivity amongst SFKs, they provide a structural framework that is consistent with, and offers a plausible basis for, the striking differences in substrate recognition observed in living cells.

The profound inability of Src to substitute for Lck in T cells, despite retaining catalytic activity toward endogenous substrates in HEK293 cells, underscores the functional specialization of closely related SFKs. Our findings suggest that subtle differences within their kinase domains are sufficient to dictate substrate selection in distinct cellular contexts, thereby enabling signaling programs that are tailored to the biological environment in which each SFK normally operates.

## 4 Materials and methods

### Antibodies and reagents

The anti-pY416-Src (diluted 1:100), anti-pY527-Src (diluted 1:50) rabbit pAbs, anti-Src rabbit pAb (1:100), anti-pSyk (Y352) (1:1000), anti-β-actin (1:2000) and anti-P-p44/42 (T202/Y204) MAPK (E10) mouse mAb (1:50) directly conjugated to AlexaFluor 647, were from Cell Signaling Technology; anti-Lck (3A5) mouse mAb (1:100), anti-CD45 (YAML 501.4) rat pAb (1:100), were from Santa Cruz Biotechnology; anti-Lck rabbit pAb (1:100) was from Novus biologicals; anti-v-Src (Ab-1) mouse mAb (1:50) and mouse anti-Phosphotyrosine (clone 4G10) (1:2000) were from Merck Millipore. Anti-pY142-ζ (K25-407.69) (1:5) coupled to AlexaFluor 647, from BD Biosciences; anti-CD3ϵ (UCHT1), used for stimulation experiments, was from Biolegend. Secondary Abs for FACS analysis and SIM were: AlexaFluor 594 donkey anti-rat IgG, AlexaFluor 488 goat anti-rabbit IgG and AlexaFluor 647 goat anti-rabbit IgG (all diluted 1:800) from Thermo Fischer Scientific. Secondary Abs for Western blotting analysis were Goat anti-mouse IRDye 680RD and Goat anti-rabbit IRDye 800CW (both diluted 1:20.000), from LI-COR Biosciences. cDNAs of Src domain-swap chimeras were custom made and purchased from Invitrogen, GeneArt^®^ Strings™ DNA Fragments.

### Generation of Tet-On inducible cell lines

Stable, inducible cell lines were generated using the Lenti-X Tet-On Advanced inducible Expression System (Clonetech) according to the manufacturer’s instructions. cDNAs of human LckWT, SrcWT and chimeric constructs were cloned into the lentiviral vector pLVX-Tight-Puro. The packaging cell line HEK293 was maintained at 37°C in DMEM complete media supplemented with10% FCS and was used for the production of recombinant lentiviruses. Specifically, 80% confluent HEK293 were transfected with a mixture of the lentiviral packaging plasmids pSVG and pSPAX2 and either pLVX-Tight-Puro containing the Lck constructs or the PLVX-Tet-On-Advanced vector driving the expression of the Tet repressor protein. 48h after transfection, the supernatants containing lentiviral particles were collected and the two batches of lentiviruses were combined and used to transduce the Lck-deficient JCaM1.6 parental cells. 48h post-infection the cells were put under selection by Puromycin (10μg/ml) and geneticin (1mg/ml) and maintained in culture at 37°C in RPMI supplemented with 10% FCS. Expression of the Lck constructs was induced by 1μg/ml Doxycycline (Sigma-Aldrich) added to the cell culture medium, routinely 24h prior to each experiment. The HEK 293 (ATCC^®^ CRL-3216) and JCaM1.6 (ATCC^®^ CRL-2063) cell lines present in this study were obtained from ATCC.

### Transient transfections

HEK293 cells were transfected at 70% confluency using a Polyethylenimine (PEI, Polysciences)-based protocol. PEIpro solution was added to 200μl of Opti-MEM (Gibco) containing the plasmids to be transfected. The mixture was incubated for 20 minutes at RT and then added dropwise to the cells. After 6 hours of incubation at 37°C, the medium was replaced and 3μg/ml of doxycycline (DOX, Sigma-Aldrich) was added to cells. Transfected cells were grown in the presence of DOX for 48 hours prior to each experiment.

### Cells, induction of Lck expression, stimulation and flow cytometry

Cell lines were cultured at a density of 300,000 cells/ml in RPMI-1640-10% FCS. Induction of protein expression was initiated by the addition of 1μg/ml Doxycycline (SIGMA) to the culture media, 24h prior to each experiment. Typically, for anti-CD3ϵ stimulation, cells were incubated with 1μg UCHT1 for 2min at 37°C. Single-cell suspensions were fixed for 10min with BD Phosflow™ Fix Buffer I (BD Biosciences) prior to 10min permeabilization with permeabilization buffer (0.5% BSA, 0.5% OmniPur Triton X-100 Surfactant (Millipore) in PBS). Primary antibodies, diluted in permeabilization buffer, were added to the cells for 1h, followed by three washes in permeabilization buffer and the addition of the corresponding secondary antibodies (again diluted in permeabilization buffer). After a final series of washes cells were analysed on a on a BD Accuri™ C6 Plus Flow Cytometer. Acquired data were analysed by the FlowJo Software (BD Biosciences). Statistical analysis was performed with Prism (GraphPad Software).

### Immunostaining, 3D-SIM, image acquisition and analysis

Single-cell suspensions were immobilized on poly-L-Lysine (Sigma-Aldrich)-coated high precision glass coverslips (Marienfeld) for 10min in a cell culture incubator. Cells were fixed for 10min with 4% Paraformaldehyde (PFA) and permeabilized with PBS/0.5% Triton-X100 for 5min. After blocking with PBS/1%BSA for 15 min, cells were stained for 1h at room temperature (RT) with the indicated primary Abs. The corresponding fluorochrome-conjugated secondary Abs were added for 1h. Nuclei were counterstained with 1 μg/ml DAPI (Sigma-Aldrich) and coverslips were mounted to microscopy slides with ProLong Gold anti fade reagent (Vectashield).

SIM was performed on an OMX V3 BLAZE microscope (GE Healthcare) using 405-, 488- and 592-nm solid-state multimode lasers and a 60x/1.42 oil UPlanSApo objective (Olympus). Multi-channel images were captured sequentially by Photometrics Cascade back-illuminated EMCCD cameras (Photometrics).1μm SIM stacks were acquired at 125nm z-distance, with 15 raw images per plane (three angles, five phases) resulting in 120 raw images in total, for each sample.

Calibration measurements of 0.2μm diameter Tetraspec fluorescent beads (Life Technologies) were used to obtain alignment parameters subsequently utilized to align images from the different color channels (OMXEditor software). A total of 120 raw image data per sample were computationally reconstructed using the SoftWoRx 6.0 software package (Applied Precision) to obtain super-resolution image stacks with a resolution of 100nm in x and y and approximately 120nm in z.

Images were converted to 16-bit average intensity composites and defined regions of interest (ROI) were segmented using binary image masks drawn according to the fluorescence obtained from the DAPI and anti-CD45 staining. The CD45 staining mask defined the PM ROI whereas and the DAPI staining mask designated the nuclear ROI. The area between the PM and nuclear masked regions was defined as the cytoplasmic ROI. Measurements of the average fluorescence intensities within the respective PM and cytoplasmic ROIs were used to calculate the plasma membrane/cytoplasmic ratios for the staining of anti-Lck, anti-Src, anti-pY416 and anti-pY505 antibodies. All image processing was performed using in-house ImageJ scripts.

### Western blotting

Cells were lysed with ice-cold lysis buffer (20 mM Tris-HCl (pH 7.5), 150 mM NaCl, 1% NP-40, 0.5% n-Dodecyl-β-D-maltoside (LM), 1 mM Na3VO4 and Protease Inhibitor Cocktail 5 (AppliChem)) for 15 min on ice. Cell lysates were centrifuged at 14,000g for 15 min at 4°C to remove cell debris. Protein concentration was measured using Bradford solution (AppliChem). Clarified lysates were boiled in Laemmli buffer at 95°C for 10 min, separated by SDS-PAGE with a 12% separation gel and then, transferred to nitrocellulose membranes (GE Healthcare). Following transfer, membranes were blocked for 1h at RT with 5% BSA diluted in TBST (0.05% Tween 20, pH 7.6) and then probed with the corresponding primary antibodies overnight at 4°C. Membranes were washed with TBST, incubated with conjugated secondary antibodies for 2h at RT and then visualised using an Odyssey F imaging system (LI-COR). Image processing was performed using the Empiria Studio Software (LI-COR).

### Molecular dynamics simulations and ensemble docking analyses

Docking calculations were performed using the PatchDock suite based on surface matching algorithm between the receptor and the ligand [1]. The kinase domains of human Lck (PDB ID:3LCK) and Src (PDB ID: 1YI6) were used as receptors, while the ITAM peptide structure was obtained from the NMR structure PDB ID: 1CV9. Initial receptor structures were used as starting structures for all-atom molecular dynamics (MD) simulations using Gromacs 2024.5 software. In particular, the two proteins were (separately) solvated in rectangular boxes large enough to contain the enzyme and 1 nm of the solvent on all sides. Sodium ions were added to provide a neutral simulation cell. During the simulations, the temperatures were maintained at 300 K using the velocity rescaling algorithm. The Amber99sb force field was used for the solvent, the water SPC model [2] was used at a standard density (1 kg/dm^3^). The Verlet cutoff scheme was used; long-range electrostatic interactions were treated by means of the particle mesh Ewald method [3, 4]. All the analyses were performed using the Gromacs tools included in the package, unless otherwise specified. The first 20 ns of the two trajectories were considered as equilibration and have been removed from the analysis (26–29).

### Statistical analysis

GraphPad Prism 9 (GraphPad Software) was the primary tool for statistical analysis. An unpaired two-tailed Student’s t test was used to determine the significance of differences, with data sets summarized as mean +/- SD. A threshold of p<0.05 denoted statistical significance (*P < 0.05, **P < 0.01, ***P < 0.001, ****P < 0.0001; ns: not significant). The reproducibility of all experimental results was validated across several independent experiments.

## Data Availability

The original contributions presented in the study are included in the article/**Supplementary Material**. Further inquiries can be directed to the corresponding author.
