# Supplementary material for: Molecular determinants of differential substrate selection between the Src family kinases Lck and Src
Source: Front Immunol. 2026 Aug 10;17:1880421. doi: 10.3389/fimmu.2026.1880421 (PMC13500599; doi:10.3389/fimmu.2026.1880421)
Supplement: Supplementary file 1 [file DataSheet1.pdf]

Supplementary Material

1 Supplementary Figures and Tables

1.1 Supplementary Figures

Supplementary Figure 1

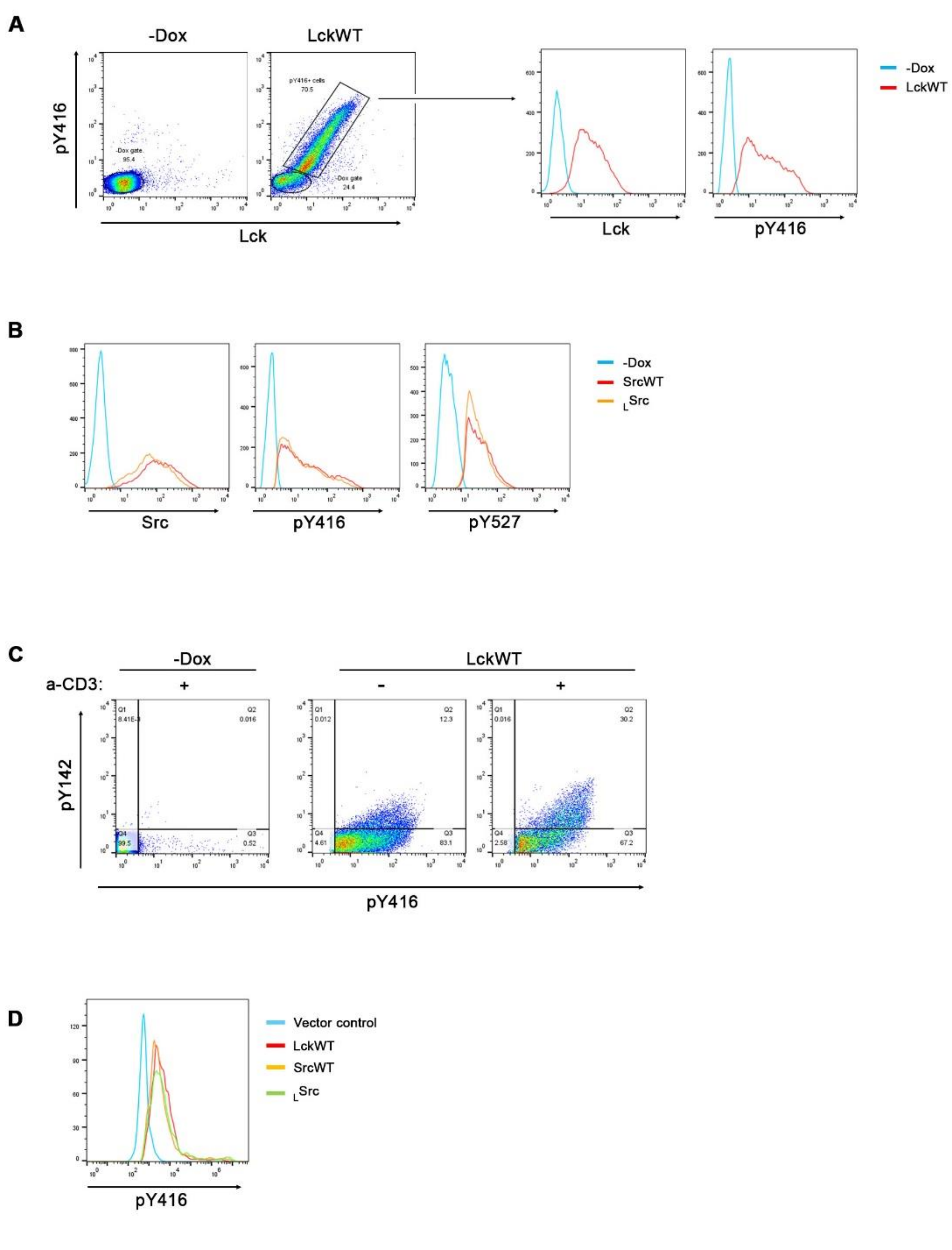

Supplementary Figure 1.

(A) Gating strategy for FACS analysis. JCaM1.6 cells transduced with the LckWT construct were cultured for 48h in the absence or presence of Dox, stained with a-Lck and a-pY416 and analysed by FACS. Single cells (from FSC-A versus FSC-H dot plots-not shown) positive for Lck/pY416 expression were selected based on gating set by respective antibodies' fluorescence in the -Dox samples. Graphs presented in the manuscript display MFI values or frequencies of cells within the $pY416^+$ subsets (or $pY527^+$ cells in the corresponding experiments). This gating strategy was followed in all FACS-based analyses throughout the study. Adjacent overlay histograms show the expression levels of Lck, and its active form as indicated.

(B) Overlay histograms of cells expressing SrcWT, $_L$Src or a mix of their -Dox counterparts stained with a-Src, a-pY416 and a-pY527.

C) Gating strategy for stimulation experiments. LckWT-expressing cells and their -Dox counterparts were incubated in the presence or absence of 1μg/μl anti-CD3ε (clone UCHT1) for 2min at 37°C. Samples were stained with a-pY142 and a-pY416 and analysed by FACS. $pY416^+$ populations (isolated as in **(A)** were further gated based on baseline fluorescence of the indicated antibodies in the -Dox sample. Percentage of cells in the upper right quadrant (Q2) represent the $pY142^+$ populations.

(D) Representative overlay histogram of HEK293 cells co-transfected with ZAP-70 and either empty vector, LckWT, SrcWT or $_L$Src, stained with a-pY416 and analyzed by FACS to verify comparable levels of active SFKs in the transfected samples. Cells used for flow cytometry were obtained from the same transfections used for the corresponding immunoblot analysis shown in Figure 1F.

Supplementary Figure 2

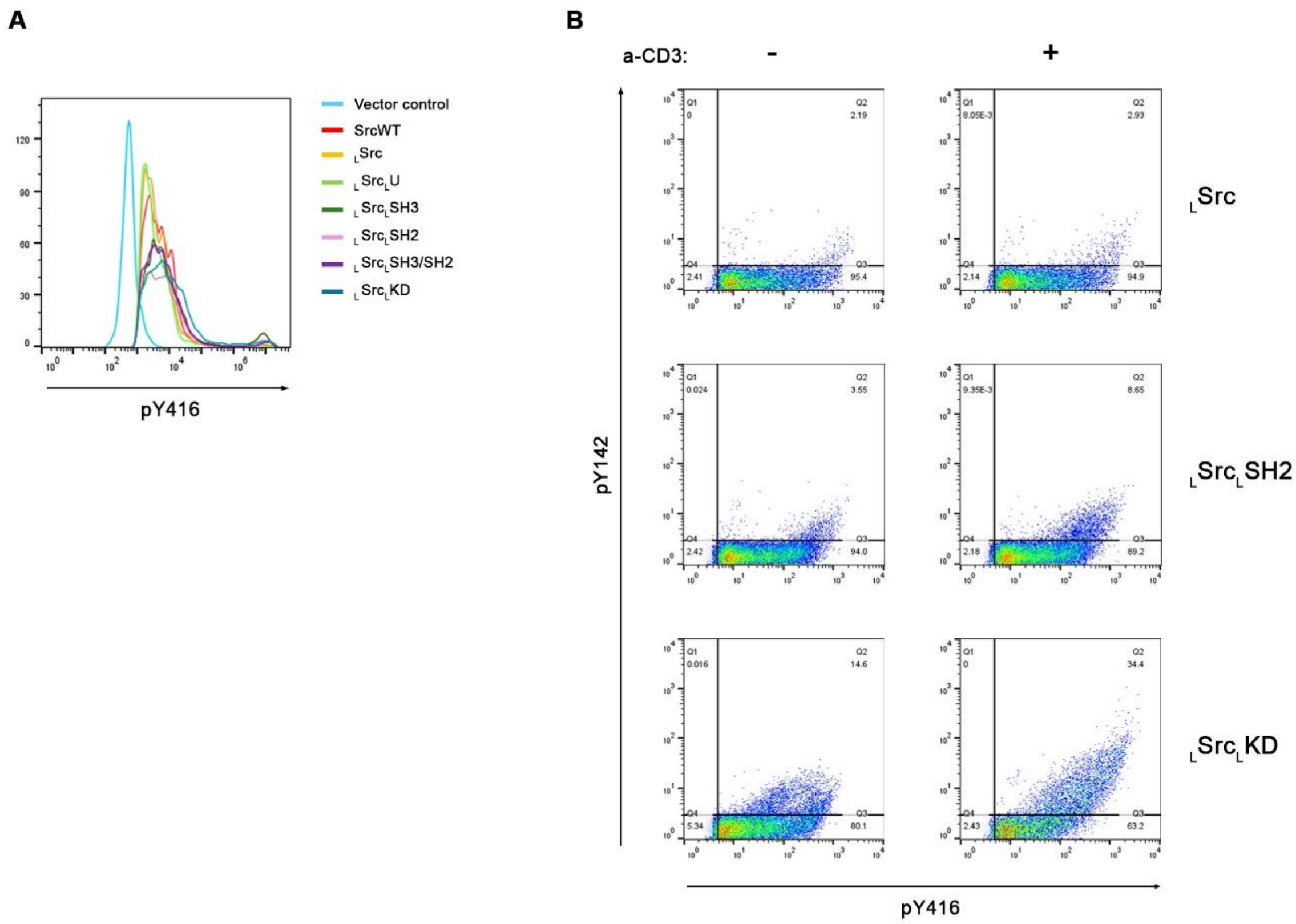

Supplementary Figure 2.

(A). Representative overlay histogram of HEK293 cells co-transfected with either empty vector, SrcWT, $_{L}$Src, or the domain-swap chimeras stained with a-pY416 and analyzed by FACS to verify comparable levels of active SFKs in the transfected samples. Cells used for flow cytometry were obtained from the same transfections used for the corresponding immunoblot analysis shown in Figure 2C.

(B) Representative 2D FACS plots of JCAM1.6 lines expressing the indicated constructs, stimulated with 1μg/ml a-CD3ε for 2min at 37ºC and stained with a-pY142 and a-pY416. Gating as in Supplementary Figure 1. Only $_{L}$Src$_{L}$SH2 is displayed as a representative profile for the swap chimeras bestowing a limited improvement on pY142 only at very high levels of SFK activity, as the rest of the constructs exhibit nearly identical patterns

1.2 Supplementary Tables

Supplementary Table 1. Design of Src domain-swap chimeras[1]

**Chimeric Protein**	**Fusion site between Src and Lck**	
	**N-terminal**	**C-terminal**
$_{L}$Src$_{L}$U	**GCGCSSHPEDDWME...**	...***GSNPPAS***PLAGGVTTF...
$_{L}$Src$_{L}$SH3	...TSPQRAGPL***QDNLVIA***	***...NFVAKAN***SIQAEEWY...
**(Lck Linker)**	...CHRLTTVC***QTQKPQK***...	...***KPWWEDE***WEIPRES...
$_{L}$Src$_{L}$SH2	...NYVAPSDS***LEPEPWF***...	...***CTRLSRP***CPTSKPQT...
**(Lck C-terminal tail)**	...YLQAFLED***FFTATEGQYQPQP***	
$_{L}$Src$_{L}$SH3/SH2	...TSPQRAGPL***QDNLVIA***...	...***KPWWEDE***WEIPRES...
**(Lck Linker)**	...CHRLTTVC***QTQKPQK***...	...***KPWWEDE***WEIPRES...
**(Lck C-terminal tail)**	...YLQAFLED***FFTATEGQYQPQP***	
$_{L}$Src$_{L}$KD	...GLAKDAWE***VPRETLKL***...	...***YLRSVLE***DYFTSTEP...

[1]In all chimeras the first 16 amino acids of human Src have been replaced by the SH4 domain of Lck. **Bold characters**: sequence of the Lck region or domain according to UniProtKB, Regular characters: amino acid sequence of Src. Conserved amino acids between Src and Lck at the borders of the fusion site of the topological domains are designated in red.
